# Identification of SARS-CoV-2 variants in indoor dust

**DOI:** 10.1371/journal.pone.0297172

**Published:** 2024-02-09

**Authors:** John Van Dusen, Haley LeBlanc, Nicholas Nastasi, Jenny Panescu, Austin Shamblin, Jacob W. Smith, Michael G. Sovic, Amanda Williams, Mikkel B. M. Quam, Seth Faith, Karen C. Dannemiller

**Affiliations:** 1 Department of Microbiology, College of Arts and Sciences, The Ohio State University, Columbus, Ohio, United States of America; 2 Genetic Counseling Program, College of Biological Sciences, University of Minnesota, Minneapolis, Minnesota, United States of America; 3 Environmental Sciences Graduate Program, The Ohio State University, Columbus, Ohio, United States of America; 4 Department of Civil, Environmental & Geodetic Engineering, College of Engineering, The Ohio State University, Columbus, Ohio, United States of America; 5 Division of Environmental Health Sciences, College of Public Health, The Ohio State University, Columbus, Ohio, United States of America; 6 Applied Microbiology Services Lab, The Ohio State University, Columbus, Ohio, United States of America; 7 Department of Chemistry and Biochemistry, College of Arts and Sciences, The Ohio State University, Columbus, Ohio, United States of America; 8 Infectious Diseases Institute, The Ohio State University, Columbus, Ohio, United States of America; 9 Institute for Genomic Medicine, Nationwide Children’s Hospital, Columbus, Ohio, United States of America; 10 Division of Epidemiology, College of Public Health, The Ohio State University, Columbus, Ohio, United States of America; 11 Section of Sustainable Health, Department of Public Health and Clinical Medicine, Umeå University, Umeå, Sweden; 12 Sustainability Institute, The Ohio State University, Columbus, Ohio, United States of America; Yale University School of Medicine, UNITED STATES

## Abstract

Environmental surveillance of pathogens underlying infectious disease is critical to ensure public health. Recent efforts to track SARS-CoV-2 have utilized wastewater sampling to infer community trends in viral abundance and variant composition. Indoor dust has also been used for building-level inferences, though to date no sequencing data providing variant-scale resolution have been reported from dust samples, and strategies to monitor circulating variants in dust are needed to help inform public health decisions. In this study, we demonstrate that SARS-CoV-2 lineages can be detected and sequenced from indoor bulk dust samples. We collected 93 vacuum bags from April 2021 to March 2022 from buildings on The Ohio State University’s (OSU) Columbus campus, and the dust was used to develop and apply an amplicon-based whole-genome sequencing protocol to identify the variants present and estimate their relative abundances. Three variants of concern were detected in the dust: Alpha, Delta, and Omicron. Alpha was found in our earliest sample in April 2021 with an estimated frequency of 100%. Delta was the primary variant present from October of 2021 to January 2022, with an average estimated frequency of 91% (±1.3%). Omicron became the primary variant in January 2022 and was the dominant strain in circulation through March with an estimated frequency of 87% (±3.2%). The detection of these variants on OSU’s campus correlates with the circulation of these variants in the surrounding population (Delta p<0.0001 and Omicron p = 0.02). Overall, these results support the hypothesis that dust can be used to track COVID-19 variants in buildings.

## Introduction

Over 769 million COVID-19 cases were reported worldwide as of August 2023 [1], resulting in millions of lives lost and substantial disruption to society. In the United States alone, it has been estimated that the pandemic has cost trillions of dollars [2–4]. After the initial emergence of the virus, additional outbreaks have been fueled by the emergence of new variants that may be more transmissible, more virulent, and/or better able to evade immunity. SARS-CoV-2 has an evolutionary rate of 1.1 x 10^−3^ substitutions/site/year [5]. RNA changes such as deletions, insertions, and recombination result in production of variants with unique and sometimes harmful characteristics [6]. Six variants of concern were discovered in the United Kingdom, South Africa, Brazil, the United States, and India between December 2020 and November 2021 [7–10]. The mutations that produce these variants can lead to changes in the virus’ pathogenesis and potentially lead to reduced efficacy of current vaccines [11]. For instance, the Delta variant has a shorter incubation period and generation time than the initial strain of SARS-CoV-2. Along with these statistics, the Delta variant has also been found to have a higher basic reproductive number, meaning more cases are expected to arise from one case of Delta compared to the previous variants [12, 13]. Delta also featured a number of mutations, including several in the spike protein, that resulted in evasion of host immunity and increased transmission [14], which allowed its rise in frequency during the second wave of COVID starting in February 2021 [1]. The Delta variant led to increased risk of hospitalization, ICU admission, and death when compared to the original variant [15]. Another variant of concern (VOC) to emerge, Omicron, was first reported in November 2021 in South Africa and Botswana [16]. This variant features mutations in the receptor binding domain that may make it more transmissible than the Delta variant [17]. Investigations have shown that immunocompromised hosts may serve as incubators for the virus, allowing for the generation of these more transmissible variants [18]. The mutations in the Omicron variant also produced an increased risk of reinfection [19, 20]. Continued surveillance of COVID-19 variants may help minimize the impact these and future variants have on communities and will benefit from sampling techniques that are more affordable and less invasive.

In times of high individual testing, laboratories typically use COVID-19 positive samples collected directly from humans to characterize variants [21, 22]. Usually these are nasopharyngeal swabs or saliva samples provided by testing sites. The acquisition of these samples is intrusive and expensive, and individual testing rates will continue to be reduced as COVID-19 becomes endemic. Fortunately, variants can also be tracked through environmental monitoring such as wastewater surveillance which is capable of tracking the virus at the sewershed level [23]. Bulk floor dust is another effective, non-invasive method for monitoring SARS-CoV-2 prevalence at the building scale [24]. Vacuumed dust is already being collected in many buildings, and PCR-based analysis can determine the presence and concentration of viral RNA from dust samples. Dust sampling can also be used to monitor for variants of concern in high-risk settings such as congregate care facilities, prisons, residence halls, and schools. This information can be used in conjunction with individual testing and wastewater monitoring at the sewershed level to conduct surveillance for COVID-19 and activate components of a risk management plan [24]. However, we need to understand if we can use building dust to monitor for variants of concern.

The goal of this paper is to present a method for detection of SARS-CoV-2 variants in vacuumed dust from buildings for ongoing monitoring. We present a combined extraction and sequencing technique as well as method detection limit information. Bulk dust offers several advantages over wastewater testing in certain situations. For instance, sample collection for wastewater can be difficult while vacuumed dust is already collected in many circumstances. Many buildings do not have a known, reliable wastewater sampling point and not all infected individuals secrete this respiratory virus in feces [25]. Additionally, wastewater requires some challenging pre-concentration steps during processing. Every building has some amount of dust that can be collected and analyzed for the presence of SARS-CoV-2 without any intense preparation prior to analysis. The data provided by dust can be used with wastewater data, complementing each other for comprehensive viral surveillance.

## Results

### RNA sequencing from dust

A range of 7.2 x 10^5^–1.32 x 10^8^ total sequencing reads were obtained from each of the 93 sequenced samples. The samples were sequenced across 13 sequencing runs that had mean Q30 scores of 94.6% (range 92.7%-96.2%). Across the samples, an average of 77.1% (±2.09%) of the SARS-CoV-2 genome was sequenced at a coverage >10x (range 1.31–98.9%; S1 Table).

### Identifying lineages in dust

**Fig 1:**
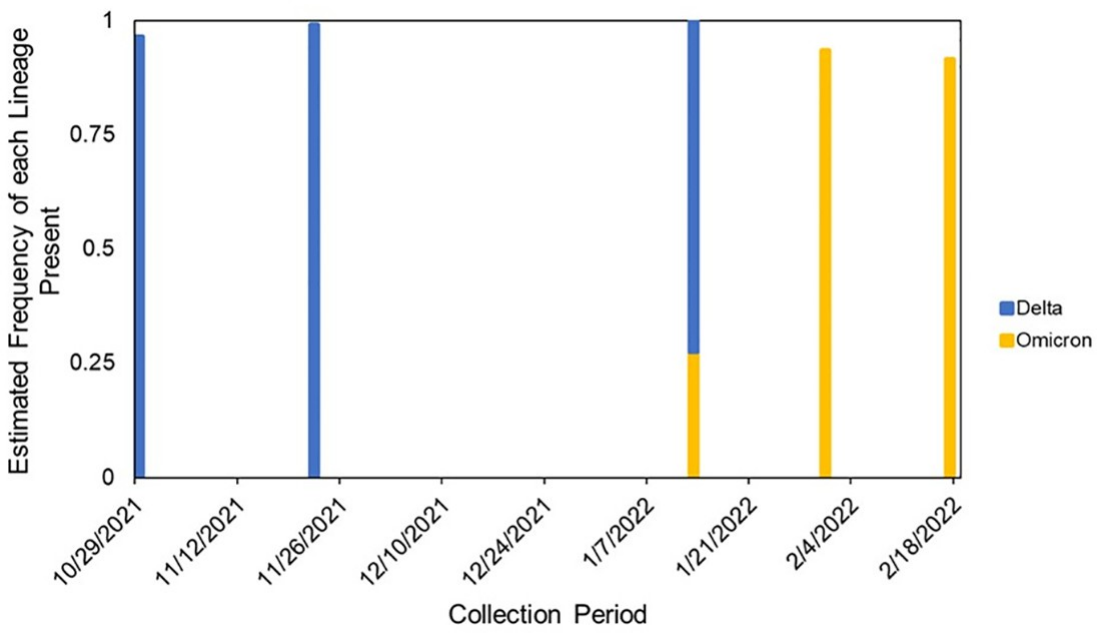
Tracking COVID-19 lineages in a single building. Samples were collected over a 5-month period with a 1-month gap due to a notable reduced campus residential population during the holidays. Each line represents one sample, and the color of the line indicates the estimated frequency of the lineage present in that sample based on measured mutations. There is a gap from November 2021 to January 2022 due to holiday break and a reduced campus residential population.

We successfully identified and differentiated between our targeted lineages from a single floor of a residence (Fig 1).

Delta was found to be the dominant strain in the building during late October and going through November. Delta was estimated to appear in samples with a frequency of 96.5% and 99.1% prior to 2022. When classes resumed on campus in January, we captured the shift in variants. Omicron was observed in this building on January 13^th^, 2022, at an estimated frequency of 27.5%. The next collection date, January 31^st^, showed a complete replacement of Delta with.

Omicron. Omicron appeared in this dust sample with an estimated frequency of 93.4% while there was no Delta detected. This dominance of Omicron continued to the next collection period where Omicron had an estimated frequency of 91.6% while Delta was not observed.

### Identifying lineages in a building from multiple floors

**Fig 2:**
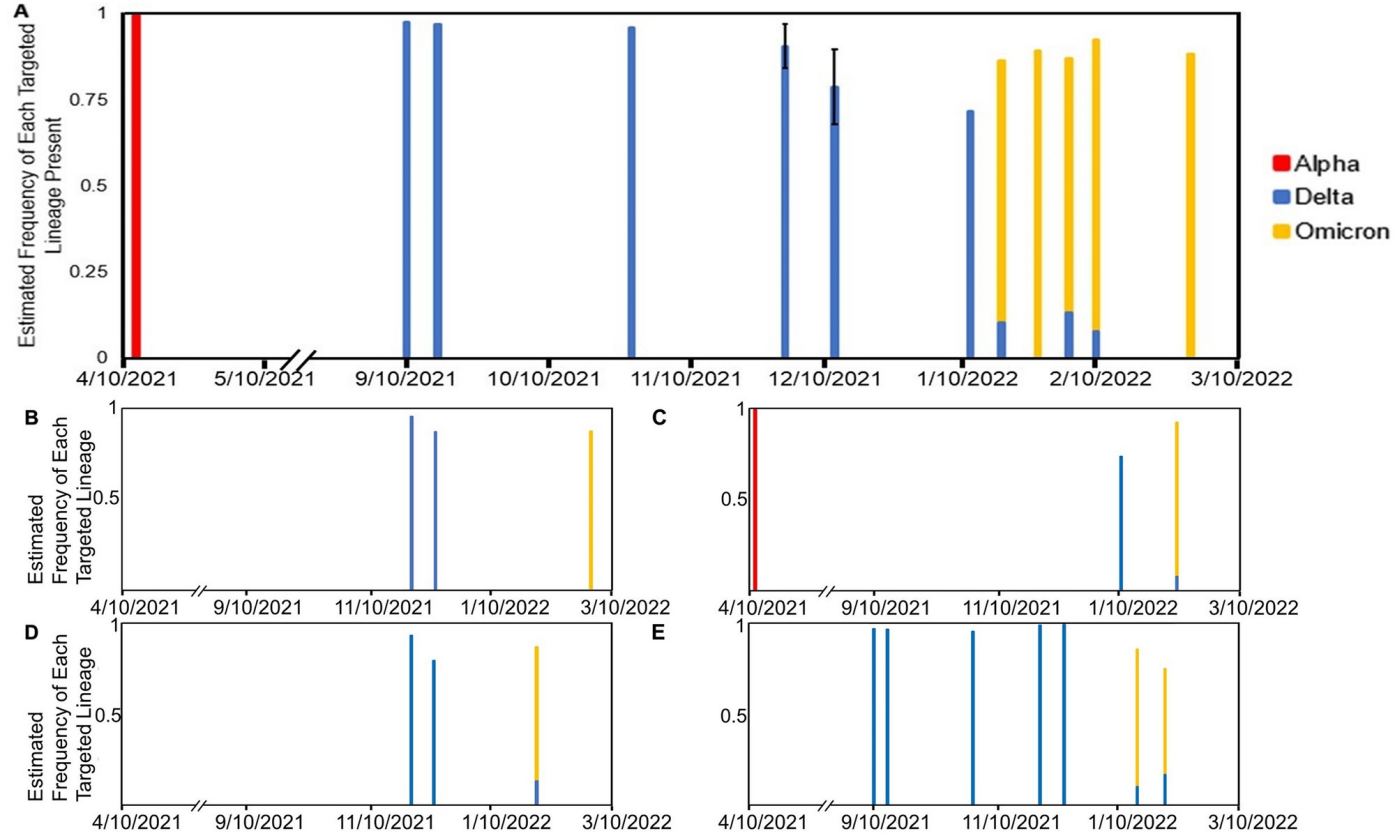
Tracking COVID-19 lineages in a public library building on a floor-by-floor level. A) The overall data for the building sampled from any floors. Samples from the same date are averaged together. Next are the floors that were sampled during the collection period: B) Floor 2, C) Floor 3, D) Floor 4, E) Floors 5–11. There is a gap in data from May 2021 to September 2021 due to the summer break and the reduced presence of residential students.

We identified variants and their prevalence in a publicly used library building across multiple floors (Fig 2).

The Alpha lineage was identified in the earliest samples with an estimated frequency of 99%. The transition to Delta was not observed due to a notable reduced campus residential population over the summer 2021 term. Samples collected from floors 5–11 in early September 2021 show that Delta appeared with an estimated frequency of 97.3%. When classes and sample processing resumed after the holiday break on January 10, the same transition between Delta and Omicron that was observed in the first building was also observed in this building. All of the floors identified Delta as the dominant lineage in this building from the Fall 2021 semester to mid-January. Across floors 3, 4, and 5–11, we see a transition from Delta to Omicron in the middle of January. When Omicron first became dominant, each of these floors had similar Omicron estimated frequencies ranging approximately from 86% to 90%. This transition to Omicron coincided with what was observed in the first residence hall building (Fig 1). Unlike the first building, we saw that Delta was still present on these floors at lower frequencies of approximately 7.5% to 10%.

### Identifying lineages in buildings throughout the entire campus

We collected samples from buildings located across the Ohio State University’s campus to measure the dominant lineages. We identified which lineages were the most prevalent in each building during sample collection as well as the transition between lineages as time progressed. Similar to our previous analyses, we were able to identify targeted lineages and track their prevalence in multiple buildings across OSU’s campus (S1 Fig). The Alpha lineage was detected on April 5^th^, 2021, our earliest sampling date, with an estimated frequency of 99%. When sample collection resumed in mid-September 2021, Delta was firmly established as the dominant lineage across campus and would remain dominant until January 2022. During this period from October 2021 to January 2022, we estimated Delta to have had a frequency of 91% (±1.3%). On January 13^th^, 2022, our first collection date that was sequenced after the end of the holiday break, Omicron was detected on campus and had already become the dominant strain. Omicron maintained this position through the remainder of the collection period. Across the period of January 2022 to March 2022, Omicron had an estimated frequency of ~87% (±3.2%). Delta was still detected in several buildings, with some only containing Delta, but it never reestablished itself as the dominant strain on campus.

### Comparing dust data to other datasets

**Fig 3:**
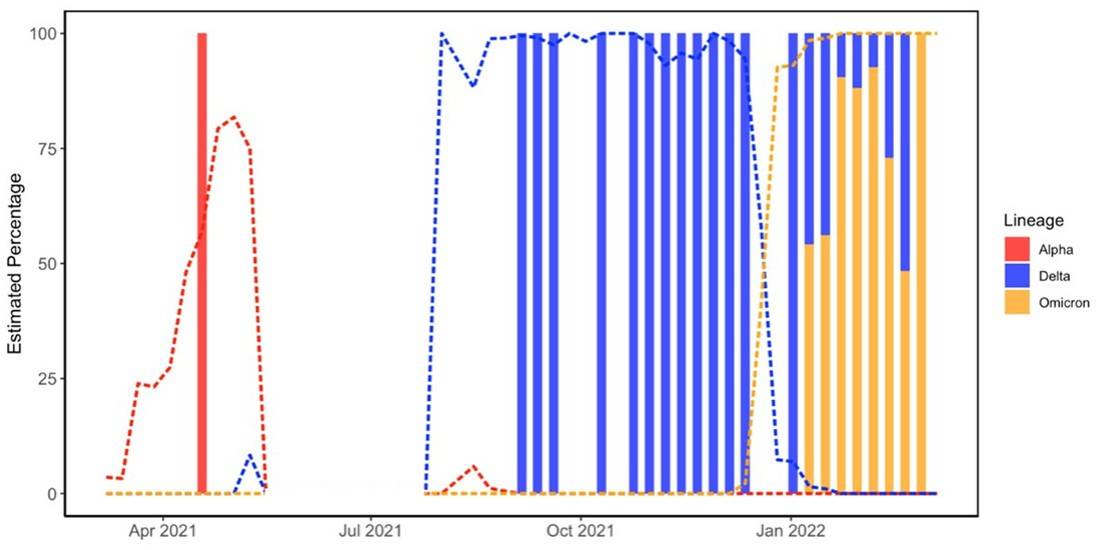
Comparison of surveillance data. Data is presented as the estimated percentage of Alpha, Delta, and Omicron from our dust sampling (bars). Dust samples were binned into weekly averages. OSU saliva surveillance (lines) is overlaid to compare the observed trends of both data sets. OSU data from January 2021 to September 2022 were used for these comparisons. There is a gap in data from May 2021 to September 2021 due to less than 5 samples being sequenced per week during the summer break and the reduced presence of residential students.

We compared the data collected from our dust samples to data obtained from OSU’s campus-wide saliva surveillance pertaining to the lineages we targeted in this study (Fig 3).

The Kolmgorov-Smiroff test with Lilliefors correction returned a p-value of 2.2 x10^-16^, indicating that the data sets were not normally distributed. The predominant strains were correlated between dust and saliva data for both Delta (Spearman rho = 0.77, p<0.0001) and Omicron (Spearman rho = 0.81, p = 0.02). In both data sets, Delta was the dominant lineage during the fall and winter of 2021. The shift from Delta to Omicron is also observed in January 2022 in both data sets. The shift in the dust data lags behind the saliva data, but this lag cannot be evaluated in this study because the sampled buildings were largely unoccupied when the shift occurred in the population and collected samples may represent deposition prior to the holiday break. Omicron was the dominant strain on campus when we completed our study in March 2022. We continued to observe some lower relative abundances of Delta in dust samples in February and March, while the OSU surveillance data did not detect Delta during this time. Future work is needed to evaluate if this is due to residual RNA or other explanations.

## Discussion

Bulk indoor dust samples can be used as an effective matrix for tracking SARS-CoV-2 variants in building populations. The methods used allowed us to identify and differentiate between the targeted lineages present in the samples and estimate their frequencies. We were also able to successfully track the transition from Delta to Omicron that occurred from December 2021 to January 2022, although we were unable to evaluate the lag time due to the holiday break and unoccupied buildings during this time.

The utilization of dust has the potential to reduce the need for expensive and invasive testing protocols. For example, vacuum bags could be examined to measure circulating variants and inform public health decisions. This information may indicate the need for interventions such as individual testing or masking. Dust sample collection is convenient and is already being done by custodial staff in many locations. Dust measurements can also be paired with wastewater tracking to ensure a more accurate analysis of areas. Wastewater treatment facilities handle waste from extremely large and highly populated areas [1, 16]. If an outbreak is identified with wastewater tracking, dust analysis can allow for more targeted responses and higher resolution information at the building level about where viruses are circulating.

Our results also demonstrate that dust can provide similar quality data to that recovered with wastewater analysis. Studies into sequencing variants from wastewater have revealed that 50% genome coverage could be achieved at Ct values ranging from 32.5 to 34.6, depending on the sequencing technique and region being targeted [26, 27]. The Ct values for dust that yielded 50% genome coverage from dust fell within this range. Wastewater samples have also been used in similar longitudinal examinations which have sought to examine the efficacy of the monitoring method at different scales. Wastewater was used to monitor SARS-CoV-2 at neighborhood level to the city-wide scale [28]. We were able to monitor the presence of SARS-CoV-2 from the campus-wide level down to individual floors in a building. We are also able to identify mutations that are characteristic of specific variants that are present in our dust samples, similar to identification of characteristic mutations in wastewater samples [29].

Comparisons of the dust data with the OSU saliva testing data have also shown that dust sampling can display similar trends as these wider testing methods. Both data sets show that Delta was the prevalent strain during late 2021 and had an estimated frequency ranging from ~75–100% on collection dates during this period. Both datasets also show that Omicron overtook Delta in January 2022 and almost completely replaced it in early 2022. Our dust data showed the persistence of Delta in buildings in February and March, despite being completely absent in the other two data sets. There are several potential explanations for why we observe this. The first is that the areas that were sampled on these dates had not been occupied since the emergence of Omicron on the campus. Between the combination of holiday break and online classes, there is the possibility that there was low foot traffic in these areas and no new viral particles were deposited. Another potential explanation could be the presence of relic RNA in the dust. This RNA could have persisted between collections and was finally collected and sequenced after the emergence of Omicron. There is also the delayed appearance of Omicron in dust sampling, with the lineage being detected in mid-January while the other datasets detected Omicron in early January. This can be explained by the campus being closed until January 10th such that that the new lineage had not been deposited into the dust yet, especially since residential students were instructed not to return to campus if they were infected and would be tested upon return (antigen self-testing upon move-in and subsequent weekly saliva-based PCR). Unfortunately we are unable to evaluate the exact lag time for the appearance of new variants in dust due to the campus buildings being largely unoccupied during the holiday break.

Dust monitoring has the potential for further applications beyond long-term COVID-19 monitoring. In addition to tracking COVID-19 variants, this tracking method could be expanded to monitor other respiratory viruses such as influenza, although verification studies are needed. Respiratory viruses can be shed by both symptomatic and asymptomatic individuals [30]. These shed viruses may be deposited in dust, enabling environmental tracking similar to SARS-CoV-2.

One of the limitations of this study is that we did not measure infectivity or viability of SARS-CoV-2 in our dust samples, though this is not needed to conduct environmental monitoring. In fact, studies on bacteriophages demonstrate that enveloped viruses are likely to have a rapid reduction in infectivity when found in dust, even though RNA persists [31]. SARS-CoV-2 RNA can still be found in vacuum bags at least 4 weeks after initial collection [24], but in real, occupied buildings dust will be removed by other processes such as vacuuming and dust resuspension. Another limitation is that dust is a non-homogeneous substrate. This can lead to variation of several orders of magnitude during sampling. To compensate for this, we implemented sieving to homogenize the dust prior to taking a sub-sample. While this method did reduce variation, it did not fully compensate for the non-homogeneity. Our collaborators in AMSL also found that taking a larger mass of dust can result in more consistent results. Another limitation of this work is that we are unable to distinguish between relic RNA from previous timepoints and recently deposited RNA. Omicron was the dominant lineage from January to March 2022, but Delta was still detected occasionally in our samples. Future work will involve investigating these remnants of Delta and determining if this was relic RNA or if Delta was still circulating in the population at low levels. Future work should also evaluate the contribution of relic RNA to measurements from real buildings. Another limitation was that there were pauses during the collection period when samples were not being collected or processed, the most notable being the gap between April and September 2021 during the transition from Alpha to Delta. Another limitation related to dating was that a number of our samples had their collection dates estimated. Custodial staff were sometimes not able to determine and report when vacuuming started or ended, meaning we estimated these dates. There is also the limitation that we only measured specific, targeted lineages. This prevented us from determining all of the variants that may have been in the dust.

Our results demonstrate that indoor dust is an effective target for monitoring variants of SARS-CoV-2 circulating in a population. Compared to other methods, such as nasopharyngeal swabs and wastewater samples, indoor dust samples are easily acquired, since they are collected during routine cleaning of buildings. On top of this, sampling indoor dust can be used to effectively monitor the presence of SARS-CoV-2 at several different scales. Specific lineages can also be identified from dust samples and the frequencies of these lineages in buildings can be estimated based on the data retrieved from those dust samples. This technique can be used in the future to help monitor for respiratory illness at the building scale.

## Materials and methods

### Sample collection

Dust samples were obtained from buildings located on The Ohio State University’s Columbus campus from April 2021 to March 2022. Maintenance staff for each building collected dust in vacuum cleaner bags as part of their normal cleaning process and marked the dates of collection. Vacuum bags were triple bagged using large zip top bags and collected on a weekly basis. The samples were labeled with the name of the building, the collection start date, and the collection end date. Depending on the vacuuming practice, interior regions or floors were also reported for some public or larger buildings. Bags were obtained from several different building types including residence halls, recreation centers, lecture halls and dining halls. Several samples were also collected from isolation rooms of COVID-19 positive individuals [24]. The vacuum cleaners located within each building were used for sample collection as part of standard custodial cleaning protocols. Vacuum interiors were not sterilized between sample collection, but vacuums were generally used for the same building week to week. Bags generally contained about 0.5–2 kg of dust, from which only approximately 50 mg was subsampled for RNA extractions. Collected bags were brought to the Applied Microbiology Services Lab (AMSL) where they were sieved using a AS200 Control Vibratory Sieve Shaker (Retsch, Haan, Germany) to 300 μm. A total of 93 vacuumed dust samples with detectable SARS-CoV-2 signal based on qPCR were obtained from custodial staff at the Ohio State University between April 2021 and March 2022. This study was determined to be “not human subjects research” by The Ohio State University Institutional Review Board.

### RNA extraction

From April 2021 to September 2021, RNA was extracted from dust samples using a Qiagen RNeasy Powermicrobiome Kit (Qiagen, Hilden, Germany). RNA extractions were performed in triplicate. The protocol was modified by combining 50 mg of dust with 65 μL 2-mercaptoethanol, 100 μL phenol:chloroform, and 650 μL of PM1 in a custom bead-beating tube. This tube contained .3g 100 μM glass beads, .1g 500 μM glass beads, and 1g garnet beads. This mixture was vortexed for 10 minutes before being centrifuged at 13000 rpm for 1 minute. All other steps of the standard protocol were followed and the included DNase1 was included. Extracted samples were either frozen at -80°C or used immediately in qPCR analysis.

During the course of the work, we continued to improve upon the techniques used for this analysis. After September 2021, RNA was extracted from dust samples using the SimplyRNA Tissue kit (AS1340, Promega Co. Madison, WI) with a Promega Maxwell RSC 48 sample concentrator with improved RNA recovery. Triplicates of 50mg of dust samples were homogenized using 2.8mm ceramic beads (OMNI International, Kennesaw GA) with 250μL of the included Homogenization Solution with 2% v/v 1-thioglycerol. This was vortexed with 250μL of the included lysis buffer and centrifuged at 4700 x *g* for one minute. Then 425μL of the supernatant was loaded into the RSC cartridge, and the included DNase was omitted. All other aspects of the manufacturer-recommended procedure were followed. Extracted samples were used immediately for qPCR analysis or stored at -80C.

### Sample screening via real-time (RT) qPCR analysis

A qPCR assay was developed for dust using N1 and RP primer probe sets recommended by the Centers for Disease Control and Prevention [32] for the detection of SARS-CoV-2 and the human RNAse-P gene. We used the IDT SARS-CoV-2 (2019-nCoV) CDC qPCR probe assay to target the N1 gene (Integrated DNA Technologies Inc, Coralville IA). The RP primer-probe set was used to determine the presence of the human RNase-P gene present in human epithelial cells. Primer and probe concentrations are detailed in Table 1. The primer-probe mix was then added to a mix containing TaqPath 1-Step Multi-Plex 4x Master Mix (Thermo-Fisher Scientific, Waltham MA), 10X Exo IPC Mix, 50X Exo IPC DNA and water following Table 2. TaqMan Exogenous Internal Positive Control reagents (Applied Biosystems, Waltham MA) were used to check for PCR inhibitors in the dust. Several wells per plate were also treated with the Exo IPC Block reagent from the TaqMan Exogenous IPC kit as a negative amplification control. Extracted RNA samples were diluted in a 1:3 dilution and plated in triplicate. Standards were plated on each plate using serial dilutions of 2019-nCOV_N Positive Control (200,000 copies/μL) (Integrated DNA Technologies, Coralville IA) from 20,000 copies/μL down to 2 copies/μL.

**Table 1:** Primer-probe mix for the qPCR analysis done on the dust samples. The per plate column corresponds to 110 wells per plate to account for reagent loss. The molar concentrations listed correspond to a total well volume of 20μL.

Reagent	Final Conc. Per Reaction	Volume per Well (μL)	Volume per Plate (μL)
2019-nCoV_N1-F (100 μM)	400 nM	0.08	8.8
2019-nCoV_N1-R (100 μM)	400 nM	0.08	8.8
2019-nCoV_N1-P (100 μM)	200 nM	0.04	4.4
RNase P-F (100 μM)	150 nM	0.03	3.3
RNase P-R (100 μM)	150 nM	0.03	3.3
RNase P-P (100 μM)	200 nM	0.04	4.4
Water	-	3.7	407.0

**Table 2:** qPCR master mix for the COVID dust assay. The per plate column corresponds to 110 wells per plate to account for reagent loss. The Exo reagents listed are part of the TaqMan Exogenous Internal Positive Control reagent kit (Applied Biosystems, Waltham MA).

Reagent	Volume per Well (μL)	Volume per Plate (μL)
TaqPath TM Master Mix	5.0	550.0
Primer-Probe-Water Mix	4.0	440.0
50X Exo IPC DNA	0.4	44.0
10X IPC Exo mix	2.0	220.0
Water	3.6	396.0

All samples were screened using RT-qPCR using an 7500 Fast qRT-PCR Thermocycler and 7500 Fast System with 21 CFR Part 11 Software (Applied Biosystems, Waltham MA). The cycling parameters for the qPCR were as follows: 52°C for ten minutes, 95°C for two minutes (denaturation) and 45 cycles of 95°C for ten seconds and 55°C for 30 seconds (annealing and extension). COVID Ct values that registered less than 34 were selected to be sequenced.

### RNA sequencing and analysis

Extracted sample RNA was reverse transcribed into cDNA (20 min 55°C, 1 min 95°C, hold 4°C) using Lunascript RT SuperMix (New England Biolabs, Ipswich, MA). Each sample was divided in half, and amplified separately in triplicate by PCR (30 sec 98°C (denaturation), (35 cycles of 15 sec 95°C then 5 min 63°C)(annealing and extension), hold 4°C) with the three amplifications per sample being pooled after amplification. The PCR amplification was performed using the ARTIC SARS_CoV-2 FS Library Prep Kit (New England Biolabs, Ipswich MA).

Pooled amplified products were cleaned using AMPure XP beads (Beckman Coulter, Indianapolis, IN) in a 0.8:1 bead-to-sample ratio, and 80% ethanol. The cleaned product was resuspended using RSB (Illumina, San Diego, CA) and prepared for tagmentation. A tagmentation master mix consisting of a 1:1 ratio of Tagmentation Buffer 1 (TB1) and Enrichment Bead-Linked Transposomes (EBLTL) (Illumina, San Diego, CA) was added to each sample and tagmentation was performed (5 min 55°C, hold 10°C). Stop Buffer (ST2) (Illumina, San Diego, CA) was added to each sample and the sample material (attached to the EBLTL beads) was separated from solution using a magnet. The supernatant liquid was removed and the beads were washed with a tagmentation wash buffer (TWB). The TWB was removed and the samples were resuspended using a PCR master mix consisting of water and Elution Primer Mix (EPM) (Illumina, San Diego, CA) in a 0.6:1 EPM-to-water ratio. Unique dual indexes (Illumina, San Diego, CA) were added to each sample and PCR was performed to bind the sample material to the indexes (3 min 72°C, 3 min 98°C, 10 cycles of (20 sec 98°C, 30 sec 60°C, 1 min 72°C), followed by 3 min 72°C and hold 10°C) The samples were then pooled and cleaned using a 1.8:1 AMPure XP bead-to-sample ratio and ethanol, and resuspended in RSB with Tween 20 (Illumina, San Diego, CA). Indexed sequencing libraries were quantified using the ProNex NGS Library Quant Kit (NG1201, Promega Co. Madison, WI). Approximately 750pM libraries were loaded on NextSeq2000 (lllumina, San Diego, CA) 2x100bp cycle sequencing runs. Sequencing results were transmitted to the BaseSpace Cloud platform (Illumina, San Diego, CA) and converted to FASTQ file format using DRAGEN (Illumina, San Diego, CA). DRAGEN COVID Lineage app v3.5.1 (Illumina, San Diego, CA) was used to align sequence data and produce quality metrics, variant calls, consensus genome sequences, and variant tables.

The R package MixviR v3.5.2 [33] allows for the deconvolution of potentially mixed microbial samples (including those that contain more than one viral variant) at both the primary and sublineage levels. We used MixviR to identify and estimate the relative frequencies of specific SARS-COV-2 lineages from each sample. The sensitivity of MixviR to detect low frequency variants is dependent upon sequencing coverage [33]. Mutations used to characterize each target viral lineage or sublineage included those that occurred in at least 75% of the samples from the given target according to the outbreak.info [34] database as of April 2022. The specific list of mutations characterizing the lineages/sublineages that was obtained from this database and used as input for the MixviR analysis, along with code for all analyses, is available at (https://github.com/mikesovic/VanDusen_etal_Dust_2023). Raw fastq sequence files used in this study have been submitted to the Sequence Read Archive (SRA) under Bioproject accession PRJNA1045708 (https://www.ncbi.nlm.nih.gov/bioproject/PRJNA1045708).

### Comparisons with other datasets

Data pertaining to saliva surveillance performed on OSU’s campus were obtained from GISAID database [35] by downloading data for samples with the prefixes “20AM-”, “21AM-”, and “22AM-”. The OSU surveillance data were collected and submitted to the database by the Applied Microbiology Services Lab (AMSL) on OSU’s campus. These OSU data represent all of the sequenced samples submitted from 2020–2022. There were 5,619 samples submitted to GISAID across these three years. These samples were binned in order to focus on WHO variants of concern for our analysis. All lineages were binned into the categories of “Alpha”, “Delta”, and “Omicron” in order to ensure accurate comparisons between all datasets. Correlations between the relative abundances of Delta and Omicron lineages between the dust samples and the saliva samples were evaluated by Spearman Rank Correlation comparison after initial tests for normality of the data with the Kolmogorov-Smirnoff test with Lilliefors correction indicated significant departures from normal distributions. All statistical analyses were conducted within RStudio with R version 4.2.0 [36].

## Supporting information

S1 Table Dust collection information. (DOCX)

S1 Fig Campus-wide monitoring of SARS-CoV-2 lineages. (TIF)

## References

[1] WHO Coronavirus (COVID-19) Dashboard [Internet]. [cited 2022 Nov 1]. https://covid19.who.int/.

[2] del Rio-Chanona RM, Mealy P, Pichler A, Lafond F, Farmer JD. Supply and demand shocks in the COVID-19 pandemic: an industry and occupation perspective. Oxf Rev Econ Policy. 2020 Aug 29;36(Supplement_1):S94–137. 10.1093/oxrep/graa033 PMC7499761 40504218

[3] Walmsley T, Rose A, Wei D. The Impacts of the Coronavirus on the Economy of the United States. Economics of Disasters and Climate Change. 2020 Dec 10;5(1):1–52. doi: 10.1007/s41885-020-00080-1 33319165 PMC7725664

[4] Macroeconomic consequences of the COVID-19 pandemic. Econ Model. 2023 Mar 1;120:106147. doi: 10.1016/j.econmod.2022.106147 36570545 PMC9768433

[5] Duchene S, Featherstone L, Haritopoulou-Sinanidou M, Rambaut A, Lemey P, Baele G. Temporal signal and the phylodynamic threshold of SARS-CoV-2’, Virus Evol, 6 (2), veaa061. 2020. 33235813 10.1093/ve/veaa061 PMC7454936

[6] Davidson AD, Williamson MK, Lewis S, Shoemark D, Carroll MW, Heesom KJ, et al. Characterisation of the transcriptome and proteome of SARS-CoV-2 reveals a cell passage induced in-frame deletion of the furin-like cleavage site from the spike glycoprotein [Internet]. Vol. 12, Genome Medicine. 2020. 10.1186/s13073-020-00763-0. PMC7386171 32723359

[7] Volz E, Mishra S, Chand M, Barrett JC, Johnson R, Geidelberg L, et al. Transmission of SARS-CoV-2 Lineage B.1.1.7 in England: Insights from linking epidemiological and genetic data [Internet]. 10.1101/2020.12.30.20249034.

[8] Tegally H, Wilkinson E, Giovanetti M, Iranzadeh A, Fonseca V, Giandhari J, et al. Emergence and rapid spread of a new severe acute respiratory syndrome-related coronavirus 2 (SARS-CoV-2) lineage with multiple spike mutations in South Africa. MedRxiv [Internet]. 2020; https://www.medrxiv.org/content/10.1101/2020.12.21.20248640v1.abstract.

[9] Kupferschmidt K, Wadman M. Delta variant triggers new phase in the pandemic. Science. 2021;372(6549):1375–6.

[10] CDC. Centers for Disease Control and Prevention. 2023 [cited 2023 Jul 18]. SARS-CoV-2 Variant Classifications and Definitions. https://www.cdc.gov/coronavirus/2019-ncov/variants/variant-classifications.html.

[11] Darby AC, Hiscox JA. Covid-19: variants and vaccination. BMJ. 2021 Mar 23;372:n771. doi: 10.1136/bmj.n771 33757984

[12] Zhang M, Xiao J, Deng A, Zhang Y, Zhuang Y, Hu T, et al. Transmission Dynamics of an Outbreak of the COVID-19 Delta Variant B.1.617.2—Guangdong Province, China, May-June 2021. China CDC Wkly. 2021 Jul 2;3(27):584–6. doi: 10.46234/ccdcw2021.148 34594941 PMC8392962

[13] Liu Y, Rocklöv J. The reproductive number of the Delta variant of SARS-CoV-2 is far higher compared to the ancestral SARS-CoV-2 virus. J Travel Med. 2021 Aug 9;28(7):taab124. doi: 10.1093/jtm/taab124 34369565 PMC8436367

[14] ASM.org [Internet]. 2021 [cited 2023 May 30]. How Dangerous Is the Delta Variant (B.1.617.2)? https://asm.org:443/Articles/2021/July/How-Dangerous-is-the-Delta-Variant-B-1-617-2.

[15] Lang K. Delta Variant has 235% Higher Risk of ICU Admission than Original Virus. Medical News Today. 2021.

[16] Del Rio C, Omer SB, Malani PN. Winter of Omicron-The Evolving COVID-19 Pandemic. JAMA. 2022 Jan 25;327(4):319–20. doi: 10.1001/jama.2021.24315 34935863

[17] Kumar S, Thambiraja TS, Karuppanan K, Subramaniam G. Omicron and Delta variant of SARS-CoV-2: A comparative computational study of spike protein. J Med Virol. 2022 Apr;94(4):1641–9. doi: 10.1002/jmv.27526 34914115

[18] Corey L, Beyrer C, Cohen MS, Michael NL, Bedford T, Rolland M. SARS-CoV-2 Variants in Patients with Immunosuppression. N Engl J Med. 2021 Aug 5;385(6):562–6. doi: 10.1056/NEJMsb2104756 34347959 PMC8494465

[19] Garcia-Beltran WF, St Denis KJ, Hoelzemer A, Lam EC, Nitido AD, Sheehan ML, et al. mRNA-based COVID-19 vaccine boosters induce neutralizing immunity against SARS-CoV-2 Omicron variant. Cell. 2022 Feb 3;185(3):457–66.e4. doi: 10.1016/j.cell.2021.12.033 34995482 PMC8733787

[20] Pulliam JRC, van Schalkwyk C, Govender N, Others. Increased risk of SARS-CoV-2 reinfection associated with emergence of the Omicron variant in South Africa. MedRixv. 2021. 2021. 10.1126/science.abn4947 PMC8995029 35289632

[21] Zhang W, Govindavari JP, Davis BD, Chen SS, Kim JT, Song J, et al. Analysis of Genomic Characteristics and Transmission Routes of Patients With Confirmed SARS-CoV-2 in Southern California During the Early Stage of the US COVID-19 Pandemic [Internet]. Vol. 3, JAMA Network Open. 2020. p. e2024191. 10.1001/jamanetworkopen.2020.24191. PMC7542329 33026453

[22] Webb LM, Matzinger S, Grano C, Kawasaki B, Stringer G, Bankers L, et al. Identification of and Surveillance for the SARS-CoV-2 Variants B.1.427 and B.1.429—Colorado, January–March 2021 [Internet]. Vol. 70, MMWR. Morbidity and Mortality Weekly Report. 2021. p. 717–8. 10.15585/mmwr.mm7019e2. PMC8118155 33988184

[23] Randazzo W, Cuevas-Ferrando E, Sanjuán R, Domingo-Calap P, Sánchez G. Metropolitan wastewater analysis for COVID-19 epidemiological surveillance. Int J Hyg Environ Health. 2020 Sep;230:113621. doi: 10.1016/j.ijheh.2020.113621 32911123 PMC7462597

[24] Renninger N, Nastasi N, Bope A, Cochran SJ, Haines SR, Balasubrahmaniam N, et al. Indoor Dust as a Matrix for Surveillance of COVID-19 [Internet]. Vol. 6, mSystems. 2021. 10.1128/msystems.01350-20. PMC8547012 33850045

[25] Larsen DA, Wigginton KR. Tracking COVID-19 with wastewater. Nat Biotechnol. 2020 Oct;38(10):1151–3. doi: 10.1038/s41587-020-0690-1 32958959 PMC7505213

[26] Izquierdo-Lara R, Elsinga G, Heijnen L, Munnink BBO, Schapendonk CME, Nieuwenhuijse D, et al. Monitoring SARS-CoV-2 Circulation and Diversity through Community Wastewater Sequencing, the Netherlands and Belgium. Emerg Infect Dis. 2021 May;27(5):1405–15. doi: 10.3201/eid2705.204410 33900177 PMC8084483

[27] Crits-Christoph A, Kantor RS, Olm MR, Whitney ON, Al-Shayeb B, Lou YC, et al. Genome Sequencing of Sewage Detects Regionally Prevalent SARS-CoV-2 Variants. MBio [Internet]. 2021 Jan 19;12(1). Available from: doi: 10.1128/mBio.02703-20 33468686 PMC7845645

[28] Longitudinal SARS-CoV-2 RNA wastewater monitoring across a range of scales correlates with total and regional COVID-19 burden in a well-defined urban population. Water Res. 2022 Jul 15;220:118611. doi: 10.1016/j.watres.2022.118611 35661506 PMC9107283

[29] SARS-CoV-2 variant detection at a university dormitory using wastewater genomic tools. Sci Total Environ. 2022 Jan 20;805:149930. doi: 10.1016/j.scitotenv.2021.149930 34536875 PMC8421076

[30] Birger R, Morita H, Comito D, Filip I, Galanti M, Lane B, et al. Asymptomatic Shedding of Respiratory Virus among an Ambulatory Population across Seasons. mSphere [Internet]. 2018 Jul 11;3(4). Available from: doi: http%3A//dx.doi.org/10.1128/mSphere.00249-18 29997120 10.1128/mSphere.00249-18 PMC6041500

[31] Nastasi N, Renninger N, Bope A, Cochran SJ, Greaves J, Haines SR, et al. Persistence of viable MS2 and Phi6 bacteriophages on carpet and dust. Indoor Air. 2022 Jan;32(1):e12969. doi: 10.1111/ina.12969 34882845

[32] Research use only 2019-novel coronavirus (2019-nCoV) real-time RT-PCR primers and probes. 2020 May 29 [cited 2023 Jul 10]; https://stacks.cdc.gov/view/cdc/88834.

[33] Sovic MG, Savona F, Bohrerova Z, Faith SA. MixviR: an R Package for Exploring Variation Associated with Genomic Sequence Data from Environmental SARS-CoV-2 and Other Mixed Microbial Samples. Appl Environ Microbiol [Internet]. 2022 Oct 26 [cited 2023 Jul 5]; https://journals.asm.org/doi/10.1128/aem.00874-22. 10.1128/aem.00874-22 PMC9680627 36286480

[34] Freitas L, Maurer-Stroh S, GISAID core and curation team, Suchard MA, Wu C, Su AI, et al. Outbreak.info genomic reports: scalable and dynamic surveillance of SARS-CoV-2 variants and mutations. 2022 10.1101/2022.01.27.22269965. PMC10399614 36823332

[35] Khare S, Gurry C, Freitas L, Schultz MB, Bach G, Diallo A, et al. GISAID’s Role in Pandemic Response. China CDC Weekly. 2021 3(49): 1049–1051. doi: 10.46234/ccdcw2021.255 34934514 PMC8668406

[36] R Core Team. R: A language and environment for statistical computing. R Foundation for Statistical Computing, Vienna, Austria. 2022. URL https://www.R-project.org/.

