# Supplementary material for: Identification of SARS-CoV-2 variants in indoor dust
Source: PLoS One. 2024 Feb 9;19(2):e0297172. doi: 10.1371/journal.pone.0297172 (PMC10857703; doi:10.1371/journal.pone.0297172)
Supplement: S1 Table — (DOCX) [file pone.0297172.s001.docx]

**S1 Table: Dust Collection Information:** The list of buildings sampled during the study. Information provided is the sample number, the building the sample came from (also indicates if samples came from different floors in the same building), the type of building, the start and end of the sample’s collection period, were these dates known or estimated, the genome coverage reported from sequencing of these samples, the VOC’s identified, the number of sublineages detected, and the highest frequency sublineage detected. Date estimations were made by setting the end date as one day before samples were collected and then setting the start date one week prior to this.

| **Sample Number** | **Location** | **Building Type** | **Collection Start Date** | **Collection End Date** | **Collection Date Known or Estimated** | **% Genome w/Coverage >10x** | **VOC’s Identified** | **Number Sublineages Detected** | **Highest Frequency**  **Sublineage(s) Detected** |
| --- | --- | --- | --- | --- | --- | --- | --- | --- | --- |
|  | Building01 | Residence | 10/21/21 | 10/29/21 | Estimated | 79.4 | Delta | 1 | AY.25 |
|  | Building01 | Residence | 11/15/21 | 11/22/21 | Known | 15.1 | Delta | 2 | AY.118 |
|  | Building01 | Residence | 01/06/22 | 01/12/22 | Estimated | 82.09 | Delta; Omicron | 3 | AY.25; BA.1.1.18 |
|  | Building01 | Residence | 01/24/22 | 01/31/22 | Known | 82.72 | Omicron | 0 | NA |
|  | Building01 | Residence | 02/11/22 | 02/17/22 | Estimated | 89.82 | Omicron | 2 | BA.1.1.18 |
|  | Building02 | Residence | 01/06/22 | 01/12/22 | Estimated | 76.39 | Delta | 2 | AY.113 |
|  | Building03 | Residence | 11/25/21 | 12/02/21 | Estimated | 89.8 | Delta | 2 | AY.25 |
|  | Building03 | Residence | 01/06/22 | 01/12/22 | Estimated | 84.22 | Delta; Omicron | 4 | AY.1; BA.1.15 |
|  | Building04 | Residence | 11/27/21 | 12/03/21 | Estimated | 89.52 | Delta | 2 | AY.47 |
|  | Building04 | Residence | 01/28/22 | 02/04/22 | Estimated | 82.75 | Delta; Omicron | 4 | AY.1 |
|  | Building04 | Residence | 02/04/22 | 02/10/22 | Estimated | 90.03 | Omicron | 0 | NA |
|  | Building05 | Dining | 02/23/22 | 03/02/22 | Estimated | 89.87 | Omicron | 4 | BA.1.1.18 |
|  | Building06 | Residence | 3/22/21 | 3/29/21 | Estimated | 82.54 | NA | NA | NA |
|  | Building06 | Residence | 3/29/21 | 4/2/21 | Estimated | 80.21 | NA | NA | NA |
|  | Building06 | Residence | 4/12/21 | 4/19/21 | Estimated | 66.8 | Alpha | NA | NA |
|  | Building07 | Residence | 10/26/21 | 10/29/21 | Known | 89.57 | Delta | 1 | AY.47 |
|  | Building07 | Residence | 02/20/22 | 02/25/22 | Known | 73.79 | Omicron | 0 | NA |
|  | Building08 | Residence | 02/18/22 | 02/24/22 | Estimated | 89.85 | Omicron | 2 | BD.1 |
|  | Building08 | Residence | 02/25/22 | 03/03/22 | Estimated | 90.23 | Omicron | 1 | BA.1.1.18 |
|  | Building09 | Residence | 11/06/21 | 11/11/21 | Estimated | 65.91 | NA | NA | NA |
|  | Building09 | Residence | 01/07/22 | 01/13/22 | Estimated | 84.39 | Omicron | 0 | NA |
|  | Building10 | Residence | 02/01/22 | 02/02/22 | Known | 90.14 | Omicron | 0 | NA |
|  | Building11 | Residence | 02/04/22 | 02/15/22 | Known | 70.83 | Omicron | 1 | BA.1.1.18 |
|  | Building11 | Residence | 02/14/22 | 02/22/22 | Known | 90.28 | Omicron | 0 | NA |
|  | Building11 | Residence | 02/21/22 | 02/28/22 | Estimated | 87.96 | Omicron | 2 | BA.1.8 |
|  | Building12 | Residence | 12/28/21 | 12/28/21 | Known | 52.28 | Delta | 1 | AY.25 |
|  | Building13 | Public Building | 01/06/22 | 01/14/22 | Estimated | 90.05 | Delta; Omicron | 3 | AY.1; BA.1.15 |
|  | Building14 | Residence | 01/06/22 | 01/13/22 | Estimated | 89.56 | Omicron | 1 | BA.1.15 |
|  | Building15 | Residence | 01/06/22 | 01/12/22 | Estimated | 89.98 | Delta; Omicron | 3 | AY.1; BA.1.1.2 |
|  | Building15 | Residence | 02/10/22 | 02/17/22 | Estimated | 90.12 | Delta; Omicron | 5 | AY.1; BA.1.8 |
|  | Building16 | Residence | 02/10/22 | 02/17/22 | Estimated | 82.69 | Omicron | 0 | NA |
|  | Building16 | Residence | 02/17/22 | 02/24/22 | Estimated | 79.94 | Delta; Omicron | 2 | AY.47; BA.1.15 |
|  | Building17 | Lecture Hall | 10/17/21 | 10/17/21 | Known | 82 | Delta | 1 | AY.44 |
|  | Building17 | Lecture Hall | 11/14/21 | 11/14/21 | Known | 54.11 | Delta | 2 | AY.44 |
|  | Building17 | Lecture Hall | 12/26/21 | 12/26/21 | Known | 1.31 | NA | NA | NA |
|  | Building17 | Lecture Hall | 01/23/22 | 01/23/22 | Known | 14.11 | NA | NA | NA |
|  | Building17 | Lecture Hall | 02/06/22 | 02/06/22 | Known | 65.01 | NA | NA | NA |
|  | Building17 | Lecture Hall | 02/13/22 | 02/13/22 | Known | 34.98 | Omicron | 1 | BA.1.1.18 |
|  | Building18 | Residence | 11/30/21 | 12/02/21 | Estimated | 90.21 | Delta | 6 | AY.47 |
|  | Building19 | Residence | 02/11/22 | 02/18/22 | Estimated | 90.15 | Omicron | 0 | NA |
|  | Building20 | Residence | 11/27/2021 | 12/03/21 | Estimated | 85.41 | Delta | 1 | AY.25 |
|  | Building21 | Residence | 11/06/21 | 11/12/21 | Estimated | 50.92 | Delta | 0 | NA |
|  | Building21 | Residence | 11/13/21 | 11/19/21 | Estimated | 89.47 | Delta | 2 | AY.119 |
|  | Building21 | Residence | 01/07/22 | 01/13/22 | Estimated | 86.81 | Omicron | 2 | BA.1.1.18 |
|  | Building21 | Residence | 01/21/22 | 01/27/22 | Estimated | 90.04 | Delta; Omicron | 4 | AY.1; BA.1.1.18 |
|  | Building21 | Residence | 01/28/22 | 02/04/22 | Estimated | 90.07 | Omicron | 1 | BA.1.1.18 |
|  | Building22 | Residence | 09/17/21 | 09/23/21 | Estimated | 98.88 | Delta | 2 | AY.3 |
|  | Building22 | Residence | 01/06/22 | 01/12/22 | Estimated | 87.01 | Delta | 1 | AY.44 |
|  | Building23 | Public Building | 01/07/22 | 01/13/22 | Estimated | 75.21 | Delta; Omicron | 1 | AY.1 |
|  | Building24 | Residence | 09/17/21 | 09/23/21 | Estimated | 95.02 | Delta | 1 | AY.47 |
|  | Building24 | Residence | 11/27/21 | 12/03/21 | Estimated | 71.96 | Delta | 1 | AY.47 |
|  | Building24 | Residence | 01/14/22 | 01/20/22 | Estimated | 90.19 | Delta; Omicron | 3 | AY.32 |
|  | Building24 | Residence | 01/21/22 | 01/27/22 | Estimated | 89.53 | Omicron | 0 | NA |
|  | Building24 | Residence | 01/28/22 | 02/04/22 | Estimated | 83.14 | Delta; Omicron | 3 | AY.1 |
|  | Building24 | Residence | 02/11/22 | 02/17/22 | Estimated | 89.53 | Omicron | 2 | BA.1.1.18 |
|  | Building25 | Lecture Hall | 11/27/21 | 12/03/21 | Estimated | 30.86 | Delta | 0 | NA |
|  | Building26 | Public Building | 10/06/21 | 10/13/21 | Estimated | 85.33 | Delta | 2 | AY.47 |
|  | Building26 | Public Building | 10/21/21 | 10/28/21 | Estimated | 74.68 | Delta | 1 | AY.47 |
|  | Building26 | Public Building | 01/14/22 | 01/20/22 | Estimated | 89.94 | Omicron | 1 | BA.1.15 |
|  | Building27 | Residence | 01/06/22 | 01/13/22 | Estimated | 51.5 | Delta | 2 | AY.24 |
|  | Building27 | Residence | 02/09/22 | 02/16/22 | Estimated | 89.86 | Omicron | 0 | NA |
|  | Building28 | Residence | 01/07/22 | 01/13/22 | Estimated | 91.51 | Omicron | 0 | NA |
|  | Building28 | Residence | 02/11/22 | 02/17/22 | Estimated | 89.66 | Omicron | 0 | NA |
|  | Building29 | Residence | 01/07/22 | 01/13/22 | Estimated | 77.87 | Omicron | 0 | NA |
|  | Building29 | Residence | 02/25/22 | 03/03/22 | Estimated | 87.47 | Omicron | 0 | NA |
|  | Building30 | Residence | 02/04/22 | 02/10/22 | Estimated | 70.71 | Omicron | 1 | BA.1.15 |
|  | Building30 | Residence | 02/18/22 | 02/24/22 | Estimated | 89.5 | Omicron | 0 | NA |
|  | Building31 | Residence | 02/02/22 | 02/02/22 | Known | 86.98 | Delta; Omicron | 3 | AY.1; BA.1.1.18 |
|  | Building31 | Residence | 02/09/22 | 02/17/22 | Known | 90.14 | Omicron | 1 | BA.1.15 |
|  | Building31 | Residence | 02/16/22 | 02/17/22 | Known | 90.32 | Omicron | 1 | BA.1.15 |
|  | Building32 | Residence | 11/06/21 | 11/12/21 | Estimated | 36.6 | Delta | 0 | NA |
|  | Building32 | Residence | 11/20/21 | 11/23/21 | Estimated | 75.72 | Delta | 1 | AY.25 |
|  | Building32 | Residence | 01/07/22 | 01/13/22 | Estimated | 87.91 | Omicron | 0 | NA |
|  | Building33 | Residence | 11/06/21 | 11/12/21 | Estimated | 19.16 | NA | NA | NA |
|  | Building33 | Residence | 11/13/21 | 11/19/21 | Estimated | 40.6 | NA | NA | NA |
|  | Building33 | Residence | 01/28/22 | 02/04/22 | Estimated | 89.88 | Omicron | 0 | NA |
|  | Building33 | Residence | 02/04/22 | 02/10/22 | Estimated | 84.43 | Omicron | 2 | BA.1.1.18 |
|  | Building34 | Residence | 12/08/21 | 12/08/21 | Known | 55.12 | Delta | 2 | AY.47 |
|  | Building35-01 | Library | 02/25/22 | 03/03/22 | Estimated | 76.04 | Omicron | 1 | BA.1.18 |
|  | Building35-02 | Library | 11/27/21 | 12/03/21 | Estimated | 76.00 | Delta | 3 | AY.118 |
|  | Building35-03 | Library | 04/05/21 | 4/12/21 | Estimated | 52.77 | Alpha | NA | NA |
|  | Building35-03 | Library | 01/07/22 | 01/13/22 | Estimated | 47.27 | Delta | 0 | NA |
|  | Building35-03 | Library | 01/22/22 | 01/28/22 | Known | 89.7 | Delta; Omicron | 2 | AY.1 |
|  | Building35-03 | Library | 02/04/22 | 02/10/22 | Estimated | 84.29 | Delta; Omicron | 2 | AY.1 |
|  | Building35-04 | Library | 11/27/21 | 12/03/21 | Estimated | 80.38 | Delta | 3 | AY.3 |
|  | Building35-04 | Library | 01/28/22 | 02/04/22 | Estimated | 89.87 | Delta; Omicron | 6 | AY.1; BA.1.18 |
|  | Building35-11 | Library | 09/03/21 | 09/10/21 | Estimated | 98.94 | Delta | 1 | AY.3 |
|  | Building35-11 | Library | 09/09/21 | 09/16/21 | Estimated | 87.42 | Delta | 1 | AY.3 |
|  | Building35-11 | Library | 10/23/21 | 10/30/21 | Estimated | 82.6 | Delta | 1 | AY.3 |
|  | Building35-11 | Library | 11/27/21 | 12/03/21 | Estimated | 57.39 | Delta | 3 | AY.118 |
|  | Building35-11 | Library | 01/14/22 | 01/20/22 | Estimated | 90.08 | Delta; Omicron | 4 | AY.1; BA.1.15 |
|  | Building35-11 | Library | 01/28/22 | 02/04/22 | Estimated | 90.13 | Delta; Omicron | 6 | AY.1; BA.1.1.18 |
|  | Building36 | Public Building | 11/01/21 | 11/19/21 | Known | 89.13 | Delta | 4 | AY.25 |
