# Supplementary figures and images for: Identification of SARS-CoV-2 variants in indoor dust
Source: PLoS One. 2024 Feb 9;19(2):e0297172. doi: 10.1371/journal.pone.0297172 (PMC10857703; doi:10.1371/journal.pone.0297172)

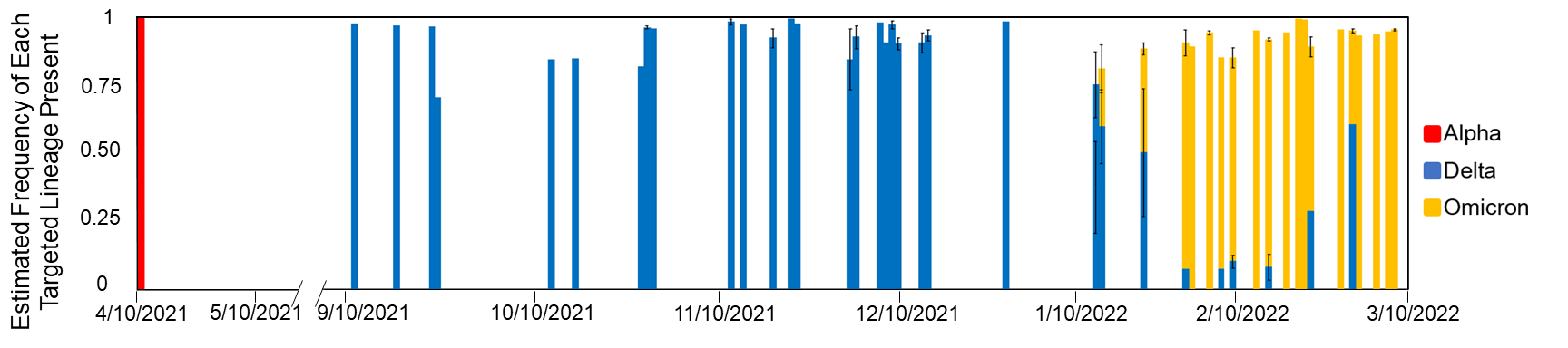

Supplement: S1 Fig — (TIF) [file pone.0297172.s002.tif]
